# plASgraph2: using graph neural networks to detect plasmid contigs from an assembly graph

**DOI:** 10.3389/fmicb.2023.1267695

**Published:** 2023-10-06

**Authors:** Janik Sielemann, Katharina Sielemann, Broňa Brejová, Tomáš Vinař, Cedric Chauve

**Affiliations:** ^1^Computational Biology, Faculty of Biology, Center for Biotechnology & Graduate School Digital Infrastructures for the Life Sciences (DILS), Bielefeld Institute for Bioinformatics Infrastructure, Bielefeld University, Bielefeld, Germany; ^2^Genetics and Genomics of Plants, Faculty of Biology, Center for Biotechnology & Graduate School Digital Infrastructures for the Life Sciences (DILS), Bielefeld Institute for Bioinformatics Infrastructure, Bielefeld University, Bielefeld, Germany; ^3^Department of Computer Science, Faculty of Mathematics, Physics and Informatics, Comenius University in Bratislava, Bratislava, Slovakia; ^4^Department of Applied Informatics, Faculty of Mathematics, Physics and Informatics, Comenius University in Bratislava, Bratislava, Slovakia; ^5^Department of Mathematics, Simon Fraser University, Burnaby, BC, Canada

**Keywords:** bioinformatics, machine learning (ML), classification, plasmids, assembly graph

## Abstract

Identification of plasmids from sequencing data is an important and challenging problem related to antimicrobial resistance spread and other One-Health issues. We provide a new architecture for identifying plasmid contigs in fragmented genome assemblies built from short-read data. We employ graph neural networks (GNNs) and the assembly graph to propagate the information from nearby nodes, which leads to more accurate classification, especially for short contigs that are difficult to classify based on sequence features or database searches alone. We trained plASgraph2 on a data set of samples from the ESKAPEE group of pathogens. plASgraph2 either outperforms or performs on par with a wide range of state-of-the-art methods on testing sets of independent ESKAPEE samples and samples from related pathogens. On one hand, our study provides a new accurate and easy to use tool for contig classification in bacterial isolates; on the other hand, it serves as a proof-of-concept for the use of GNNs in genomics. Our software is available at https://github.com/cchauve/plasgraph2 and the training and testing data sets are available at https://github.com/fmfi-compbio/plasgraph2-datasets.

## 1. Introduction

Plasmids are mobile genetic elements that are involved in horizontal gene transfer and have been shown to be a major vector for the spread of antimicrobial resistance (AMR) genes (Partridge et al., 2018; De Oliveira et al., 2020). Plasmids are extra-chromosomal DNA molecules, often circular and significantly shorter than bacterial chromosomes, and can occur in multiple copies in a bacterial cell. Whereas some bacteria do not contain any plasmid, it is common to observe several plasmids co-existing within a bacterial cell, often with different copy numbers. Due to their high mobility and impact in AMR spread, the detection of plasmids from sequencing data is an important question in One-Health epidemiologic surveillance approaches (see Cox et al., 2021).

Given sequencing data, either from a bacterial isolate or from a metagenome, the detection of plasmids can be approached at various levels of detail. The most elementary task, *contig classification*, aims at detecting which assembled contigs likely originate from a plasmid. *Plasmid binning* aims at grouping contigs into groups likely to originate from the same plasmid. Finally, *plasmid assembly* aims at reconstructing full plasmid sequences. While obtaining full plasmids provides the most accurate information, the ability to extract plasmid contigs from assembled sequencing data (the contig classification problem) already provides very useful information, allowing, for example, to identify genes that might be susceptible to transfer to other bacteria. Moreover, the prediction of plasmid contigs can be used as an input for plasmid binning or assembly. For example, the plasmid binning method gplas (Arredondo-Alonso et al., 2020) relies on a preliminary contig classification obtained with mlplasmids (Arredondo-Alonso et al., 2018), and the metagenome plasmid assembly method SCAPP (Pellow et al., 2021) relies on classifying contigs using PlasClass (Pellow et al., 2020).

While the analysis of plasmids from sequencing data has been a very active research area, the problems mentioned above are still challenging, especially when sequencing data are provided in the form of Illumina short reads (Arredondo-Alonso et al., 2017). In the present study, we propose a novel method for the contig classification problem, specifically designed to analyze short-read contigs from a single bacterial isolate.

### 1.1. Background

There exists a large corpus of algorithms for the contig classification problem, most of them developed recently. These methods rely mainly on machine learning approaches. The earliest method for contig classification was cBar (Zhou and Xu, 2010), which introduced the use of the *k*-mer profile of a contig as the main feature in a machine learning classification model; in cBar, the model was trained on a large data set of closed bacterial genome assemblies. The general principle of using *k*-mer properties as classification features has also been used in several recent machine learning classifiers, namely, PlasFlow (Krawczyk et al., 2018), mlplasmids (Arredondo-Alonso et al., 2018), and PlasClass (Pellow et al., 2020). PPR-Meta (Fang et al., 2019) is a deep learning method that relies on one-hot encoded contig sequences. PlasForest (Pradier et al., 2021) and Deeplasmid (Andreopoulos et al., 2021) are two recent methods based on machine learning models that use different features for a given contig, such as its GC content (generally plasmids have a GC content different from chromosomes) and the presence of plasmid-specific sequences, detected through the mapping against a reference plasmid database. RFPlasmid (van der Graaf-van Bloois et al., 2021) combines both types of features, the *k*-mer profile and plasmid-specific sequences. Finally, Platon (Schwengers et al., 2020) relies on a deterministic decision workflow based on a statistical score in terms of homology search against a large database of plasmid proteins further refined by considering higher level plasmid contig features. Among the methods introduced above, both mlplasmids and RFPlasmid are species-specific methods, i.e., require a model to be trained per bacterial species; in contrast, PlasFlow, PlasClass, PlasForest, and Deeplasmid are tools that do not target specific species.

The recent method 3CAC (Pu and Shamir, 2022) introduced the idea that the classification of a contig can be improved from the knowledge of the classification of the neighboring contigs in the *assembly graph*. Several tools used for assembling bacterial genomes (Bankevich et al., 2012; Wick et al., 2017; Souvorov et al., 2018) output an assembly graph containing final contigs as nodes and possible connections between them supported by sequencing data as edges. Individual molecules, such as chromosomes or plasmids, ideally correspond to walks in this graph, but some edges may be missing, disconnecting the walk. Conversely, the walks for individual molecules often form complicated tangled structures joined at shared and repeated sequences. Nonetheless, adjacent nodes often share the same molecule of origin and thus the same class. 3CAC applies simple heuristics to improve machine learning predictions for individual contigs based on their adjacency in the graph. Our aim is to integrate the information from the assembly graph directly into a machine learning model for the contig classification problem.

### 1.2. Contribution overview

Here, we introduce a novel machine learning method, plASgraph2, for the problem of classifying short-read contigs. Our method is based on combining features of existing methods with a novel approach incorporating a graph neural network (GNN) (Grattarola and Alippi, 2021). Moreover, plASgraph2 is a *de novo* tool that does not require the comparison of the input contigs with a database of known plasmids, which is of interest, for example, the analysis of samples from poorly sampled bacterial species. More precisely, plASgraph2 characterizes each contig of a bacterial genome assembly using a set of features that have been shown to differentiate plasmids and chromosomes: read coverage, used as a proxy of copy number, GC content and contig length, together with two novel features, the node degree in the assembly graph and the similarity between the contig *k*-mer profile and the whole assembly *k*-mer profile. The rationale behind using the similarity to the assembly-wide profile, rather than learning a species-specific *k*-mer profile (as done in mlplasmids and RFPlasmid), is to allow our model to be species-agnostic and avoid the necessity of training a new model for every species. Based on these features, plASgraph2 trains a GNN model whose core is a set of graph convolutional layers aimed at propagating the information from neighboring contigs in the assembly graph. The output of plASgraph2 is a pair of scores for each graph node, a plasmid score and a chromosomal score, used to determine if a given contig is likely to originate from a plasmid or a chromosome or both. Unlike other methods, the two scores associated with a contig allow to detect *ambiguous* contigs that have shared sequences of both plasmidic and chromosomal origins.

To the best of our knowledge, plASgraph2 is one of the first methods that applies GNNs to contig classification in an assembly graph, building on the idea (introduced in 3CAC) that information from neighboring contigs can improve accuracy. Outside of classification, GNNs were also used recently on assembly graphs for metagenomic contigs binning (Lamurias et al., 2022).

We trained plASgraph2 on data from the ESKAPEE group of pathogens (*Enterococcus faecium, Staphylococcus aureus, Klebsiella pneumoniae, Acinetobacter baumannii, Pseudomonas aeruginosa, Enterobacter* spp., and *Escherichia coli*) which is of primary importance in a public health setting (Partridge et al., 2018) and evaluated its performance on data sets of ESKAPEE samples and non-ESKAPEE samples. Our results show that plASgraph2 either outperforms or is comparable to the state-of-the-art methods, including species-specific methods and methods relying on databases of known plasmids.

## 2. Methods

### 2.1. Input features

The input to our problem is the assembly graph of a bacterial isolate in which nodes correspond to contigs and edges correspond to contig adjacencies supported by sequencing data. As an input to the classification task, each contig is characterized by six features as follows:

1. The *degree* of the corresponding node in the assembly graph;
2. The *contig length* divided by 2 million (so that it has a similar scale as other features);
3. The *logarithm of the contig length*;
4. The *relative GC content* defined by subtracting the average GC content (a value between 0 and 1) of the whole assembly from the contig GC content;
5. The *relative coverage* defined as the contig read depth divided by the weighted median read depth over all contigs in the assembly (weighed by contig lengths);
6. The *relative pentamer content* defined as the dot product 〈*p, q*〉 between vectors *p* and *q* representing the pentamer profile of the contig and the pentamer profile of the whole assembly, respectively. For a set of contigs *S* (which may include a single contig or all contigs in the assembly), we define #(*k, S*) to be the number of occurrences of pentamer *k* and its reverse complement in *S*. If $Z=\underset{k}{\sum }\left(\#\left(k,S\right)+\varepsilon \right)$, the pentamer profile of set *S* is simply a vector of values [(#(*k, S*)+ε)/*Z*]_*k*_; here, ε is a pseudocount and we use ε = 0.01.

The motivation to rely on relative features instead of absolute features is to enable the model to generalize across species and thus to not be dependent on species-specific values. For example, using the actual GC content as a feature would allow the model to learn that chromosomal sequences have a specific GC content and plasmid sequences also have a specific GC content. This type of knowledge is not transferable between species, as each species has a different GC content. On the other hand, the use of a relative GC content allows the model to learn that chromosomal sequences will have a GC content similar to the overall sample GC content (since chromosomal contigs dominate the assembly in length), whereas plasmid contigs will typically differ in GC content from the overall sample. This type of knowledge is more transferable between species. Regarding the relative pentamer content, one can expect that chromosomal contigs will have large values because their *k*-mer frequencies are close to those of the sample as a whole, while plasmid contigs will exhibit values closer to zero. By using the relative pentamer content, we expect that our model will be less susceptible to learning to classify chromosome sequences by simply recognizing the pentamer frequency characteristics for the chromosomes of a particular species or a clade.

### 2.2. Model architecture and training

We solve the classification task using a deep neural network model designed specifically for graph-structured data, *graph neural network* (GNN) (Kipf and Welling, 2016), with the aim to leverage the information provided by the assembly graph. The propagation of information between individual nodes is accomplished by *graph convolutional layers* (GCLs). In brief, the input to a GCL contains a vector of *k* features for each of the *n* nodes of the graph and the adjacency matrix of the graph. The layer first combines the feature vectors corresponding to the node and its neighbors, with the weight of nodes depending on their degree. The feature vector of each node is, then, transformed by a fully connected layer with ℓ output features followed by a non-linear activation. More precisely, if we organize the *n* feature vectors into an *n* × *k* matrix *X*, the graph convolutional layer can be expressed as follows:


(1) $$\begin{array}{l} Z=\sigma \left({\tilde{D}}^{-1/2}\tilde{A}{\tilde{D}}^{-1/2}X\text{Θ}+b\right), \end{array}$$

where Ã is the graph adjacency matrix with one along the diagonal, $\tilde{D}$ is a diagonal matrix where $\tilde{D_{ii}}=\underset{j}{\sum }{Ã}_{ij}$, Θ is a *k* × ℓ matrix of trainable weights, *b* is trainable bias vector of length ℓ, σ is a non-linear activation function, and *Z* is the *n* × ℓ matrix of output feature vectors. A single GCL integrates information from the immediate neighborhood of a node; by employing *d* GCLs one integrates the information from nodes at distance at most *d* for each node.

**Figure 1:**
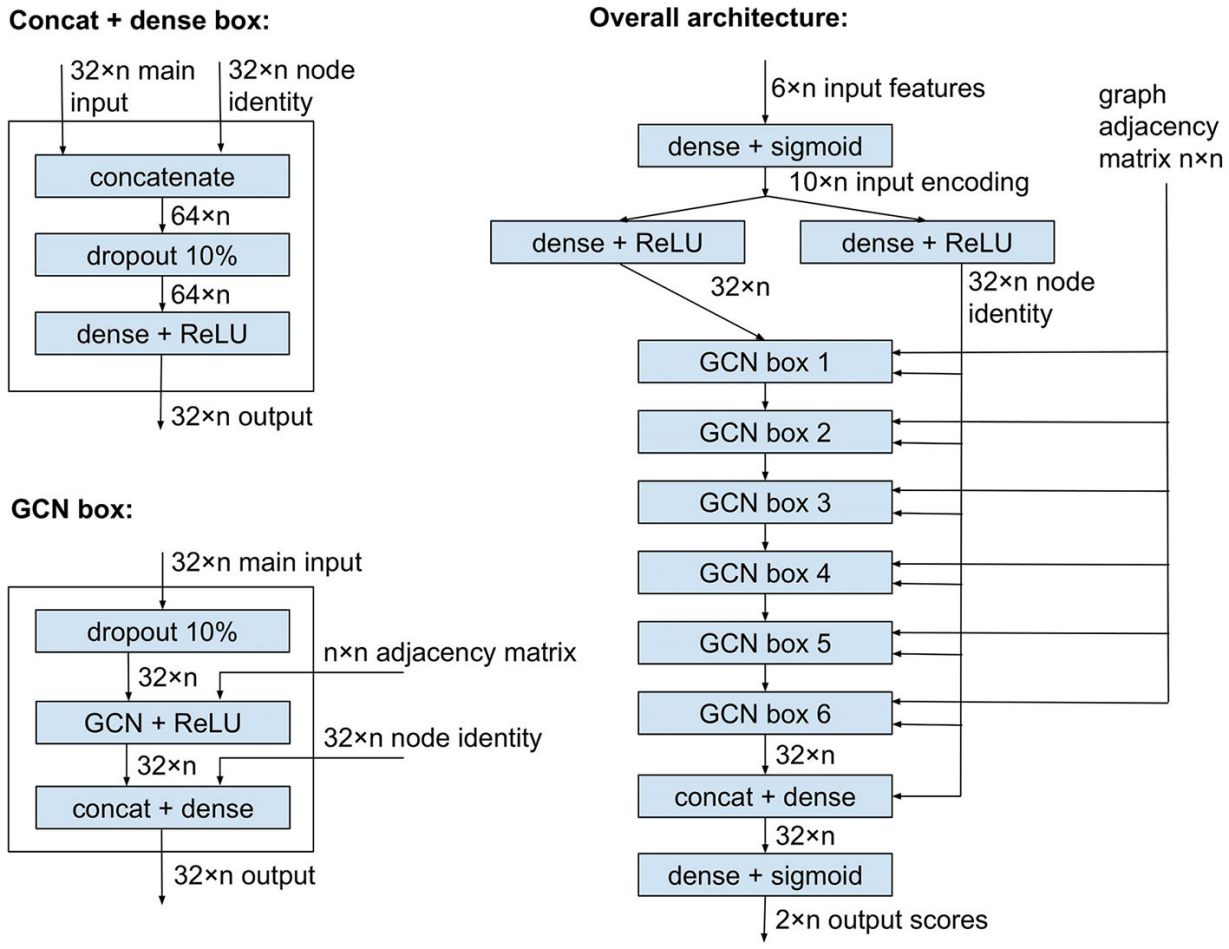
Model architecture of plASgraph2. The model takes as input the assembly graph structure and six features per node (contig). The core of the network is composed of six graph convolutional layers. The model generates two outputs per node, which facilitate the classification of plasmids and chromosomes as two separate classification tasks.

Figure 1 shows the plASgraph2 architecture. The six input features for each node are first transformed by two fully connected layers to a vector of length 32 per node. This is followed by six GCLs using the same weight matrix Θ. The last two fully connected layers operate on each node separately, finally producing two output scores, loosely interpretable as probabilities of the node being part of a chromosome and plasmid, respectively. Since these two outputs correspond to two separate classification tasks, we do not require these two scores to sum to one.

GCLs combine features of each node with features of the neighbors, and over time, the influence of the original features of a node is greatly diminished. In our task, the original features can be highly informative, especially for nodes corresponding to longer contigs; therefore, we want to maintain the node identity (original features) throughout the computation. To accomplish this, each GCL is followed by another dense layer which receives an additional input vector of length 32 for each node, representing a separate encoding of the original input features for the node. This node identity is also an input to the penultimate dense layer of the whole network.

As shown in Figure 1, the network uses ReLU and sigmoid activation functions. It also uses dropout layers to prevent overfitting. The network is trained using Adam optimizer (Kingma and Ba, 2014) with binary cross entropy loss function, a constant learning rate of 0.005, and a split of 80% of data for training and 20% for validation. The model is implemented using Keras (Chollet, 2015) and TensorFlow v2.8.0 (Abadi et al., 2015), with GCLs from Spektral v1.0.8 (Grattarola and Alippi, 2021). The number of GCLs and several other settings were chosen by exploring various values on a data set used solely for designing the architecture but disjoint with test sets used for the final evaluation.

### 2.3. Classification

Since plASgraph2 was designed to model existence of ambiguous contigs by including separate plasmid and chromosomal classification tasks, we evaluate the prediction performances for each of these tasks separately. A contig is predicted as a chromosome if the chromosome score output of the plASgraph2 model is at least 0.5 and the plasmid score is below 0.5. Similarly, it is predicted as plasmid if the plasmid score is at least 0.5, and the chromosome score is below 0.5. It is predicted as ambiguous if both scores are at least 0.5 and is unlabeled if both scores are below 0.5.

However, threshold 0.5 is arbitrary, and training the outputs using the binary cross entropy loss function does not guarantee a good balance between the precision and recall measures. Therefore, plASgraph2 provides an optional phase, switched on by default, which, after training the network, uses the validation set to adjust the threshold for each of the two classifiers. Namely, we sort all scores produced by a particular classifier on the validation set and consider the mean of each of the two distinct successive scores as a potential threshold. We choose the threshold that achieves the best F1 score on the validation set. When the trained model is applied to new data, we transform the output scores of the neural network by a piece-wise linear function so that the selected threshold is mapped to value 0.5 and endpoints 0 and 1 map to themselves. After this transformation, we can apply the original threshold 0.5 on the output scores. Nonetheless, users may choose to apply more conservative thresholds if they are interested only in high reliability predictions.

### 2.4. Training and testing data preparation

#### 2.4.1. Species

Most methods for classifying contigs target a very wide range of bacteria, while a few others, such as mlplasmids (Arredondo-Alonso et al., 2018), train species-specific models. In this study, we trained plASgraph2 on data from the ESKAPEE group of pathogens from a variety of sources (Arredondo-Alonso et al., 2018; Hikichi et al., 2019; Magalhães et al., 2019; Chan et al., 2020; Peter et al., 2020; Ono et al., 2021; Shaw et al., 2021; Acman et al., 2022; Boostrom et al., 2022) to avoid confounding factors linked to the way the sequence data were generated (see overview in Supplementary Table 1). The independent ESKAPEE test samples and non-ESKAPEE single-species data (Johnson et al., 2015; Robertson and Nash, 2018; Matsumoto et al., 2019; Chen et al., 2020; Kiesewalter et al., 2020; Shaw et al., 2021; Furuta et al., 2022; Morita et al., 2023) were then used for the comparison against other tools.

#### 2.4.2. Assembly

As plASgraph2 analyzes the assembly graph of a bacterial isolate, the method used to obtain this assembly graph is a possible confounding factor of the training data. In our experiments, every sequencing data set was assembled using both Unicycler v0.5.0 (Wick et al., 2017) and SKESA v2.4.0 (Souvorov et al., 2018), two widely used assemblers for bacterial genomes that provide an assembly graph, thus leading to two data sets per isolate. Both Unicycler and SKESA provide a read depth per contig, which was used to define the relative coverage feature.

#### 2.4.3. Ground truth contigs labeling

Once the training data set has been assembled, its contigs are required to be labeled as being either chromosomal, plasmidic, or ambiguous. This task is usually performed by assuming that a closed and annotated genome assembly is available for each training sample, which is not always the case as most genomes available in public databases are provided in the form of a contig assembly; moreover, the annotation of plasmids in closed genomes often relies on a plasmid database for the corresponding species, which introduces a potential bias.

To circumvent these issues and assess the performances of plASgraph2 and other methods in a setting where a sufficient number of closed annotated genomes are not available, we relied on the growing trend to sequence bacterial isolates using both Illumina accurate short reads and Oxford Nanopore or Pacific Bioscience noisier long reads (*hybrid sequencing*). All the samples in our training and testing data sets were sequenced using the hybrid approach.

To label the data, we first followed the general methodology introduced by mlplasmids (Arredondo-Alonso et al., 2018). First, a hybrid assembly is created using both short and long reads. This assembly is typically highly contiguous and can be easily labeled. Subsequently, a short-read assembly used for both training and testing is labeled based on homology with the hybrid assembly.

In hybrid assemblies created by Unicycler, the ground truth labels were determined primarily based on the contig length and circularity: all contigs longer than a threshold (in our experiments, we chose 1 Mbp) are labeled as “chromosome”, while shorter circular contigs are labeled as “plasmid”. The remaining short linear contigs, that can possibly be a part of an unfinished plasmid or chromosome, remain unlabeled.

To further improve classification, we used minimap2 (Li, 2018) to map the individual contigs to a set of closed genome reference sequences (Supplementary Table 2) and the curated PLSDB plasmid database (Schmartz et al., 2022). Previously unclassified contigs longer than 1,000 bp were labeled as plasmids if they mapped to the plasmid database on at least 80% of their length and did not map to the closed genome reference on more than 20% of their length. Similarly, contigs longer than 100 kbp which mapped to the closed genome reference on more than 80% of their length, but mapped to the plasmid database on at most 20% of their length, were labeled as chromosome. Conversely, previously labeled contigs were changed to unlabeled if the homology information contradicted the original labels. This included plasmid contigs with longer homology to the genome reference than to the plasmid database and chromosome contigs with longer homology to the plasmid database than to the genome reference. Finally, several samples contained PhiX bacteriophage, commonly used as a control in Illumina runs; any labels assigned to contigs corresponding to the PhiX bacteriophage have been removed.

The ground truth labels for short-read contigs were determined by mapping the short-read contigs of a sample to the corresponding hybrid assembly contigs, from which they inherit the labels. The mapping was performed using minimap2 v2.24 (Li, 2018), with -c option for accurate alignment. The key difference between our pipeline and mlplasmid method is that if a contig matches equally well to both chromosomal and plasmidic hybrid contigs, it is labeled as “ambiguous”. We have observed that without considering such ambiguous matches, the short-read assembly graphs often contained paths with nodes labeled by alternating classes, which is clearly inconsistent labeling, and the introduction of ambiguous labels allows us to avoid such artifacts. Short-read contigs matching an unlabeled hybrid contig were left unlabeled, and samples that contained more than 5% of unlabeled short-read contigs were discarded from further analysis. Supplementary Table 1 shows the statistics of the short-read contig labels in our data sets.

#### 2.4.4. Handling short contigs

Most contig classification tools exclude very short contigs from their analysis because they can not be labeled reliably. For example, mlplasmids excludes contigs of length below 1 kbp. For training and prediction, all contigs shorter than 100 bp were removed from the short-read assembly graphs, and their neighbors were connected by direct edges as part of the feature extraction process. Thus, plASgraph2 is not classifying contigs shorter than 100 bp.

## 3. Results

We evaluate the performance of plASgraph2, trained on a data set of samples from ESKAPEE species as described above, on two testing data sets: one composed from ESKAPEE samples and one composed from non-ESKAPEE samples. We compare the perfomance of plASgraph2 with a variety of plasmid classification tools, such as Deeplasmid (Andreopoulos et al., 2021), mlplasmids (Arredondo-Alonso et al., 2018), PlasClass (Pellow et al., 2020), PlasForest (Pradier et al., 2021), Platon (Schwengers et al., 2020), and RFPlasmid (van der Graaf-van Bloois et al., 2021).

### 3.1. Evaluation metrics

For each testing sample, we created two short-read assemblies using Unicycler and SKESA, removed contigs of lengths ≤ 100 bp, and obtained ground-truth labels from the corresponding hybrid assemblies as described in Section 2.

The true and predicted labels induced the counts of true positives (*TP*), true negatives (*TN*), false positives (*FP*), and false negatives (*FN*) for each classification task (plasmid and chromosome). Each contig was counted as a single unit, regardless of its length. Contigs without a ground-truth label were not included in the evaluation. Note that, contigs whose ground truth label is “ambiguous” are considered positives for both plasmid and chromosome classification tasks. As plASgraph2 can assign an “ambiguous” label to some contigs (those with score >0.5 in both classification tasks, see Section 2), such contigs were labeled both as plasmid and chromosome in our evaluation.

For each classification task, we evaluate several performance measures, including precision *TP*/(*TP*+*FP*), recall *TP*/(*TP*+*FN*), F1-score (the harmonic mean of precision and recall), and accuracy (*TP*+*TN*)/(*TP*+*FP*+*TN*+*FN*). For methods that assign scores or probabilities to contigs, the result of the classification is highly dependent on chosen score thresholds; we also consider an accuracy measure that is independent of these thresholds, the area under the receiver operating characteristic curve (AUROC). For methods that do not produce a numerical value for each contig (PlasForest abd Platon), we cannot compute the AUROC statistics. Note that, the precision and recall are undefined when the denominator is zero, and the F1 score is undefined if either precision or recall is undefined. We evaluate each of these measures on each assembly included in the testing set (where possible) and report the median value. All of the tools that we compare exhibited a large variance in accuracy of their predictions between individual samples. The median values were chosen since the resulting measures are less affected by outliers.

### 3.2. Evaluation on the ESKAPEE data set

The ESKAPEE testing set consist of 224 short-read assemblies derived from 112 isolates; each data set was assembled by both Unicycler and SKESA. The data set contains 38,110 contigs with known label longer than 100 bp and 15,687 contigs longer than 1,000 bp.

Table 1A shows that plASgraph2 performs as the best tool on the plasmid classification task under F1, accuracy, and AUROC measures. For the prediction of chromosomal contigs (Table 1B), Platon shows the highest median F1-score of 0.973, whereas plASgraph2 has the second highest median F1-score of 0.968. Supplementary Figure 1 shows the full distribution of F1 scores over individual samples. In general, it is expected that tools relying on homology will achieve better results than those relying on sequence-derived features alone. Interestingly, plASgraph2 (using sequence-derived features) can outperform homology-based tools in plasmid classification and compete with them in chromosome classification. The lack of homology information is compensated by pooling information from neighboring contigs in an assembly graph.

**Table 1:** Performance of contig classification algorithms on the ESKAPEE testing set.

**Method**	**SS**	**DB**	**AUROC**	**Precision**	**Recall**	**F1**	**Accuracy**
**A: Plasmid classification, contigs** >**100 bp**, *n*=**38,110**							
plASgraph2	–	–	**0.991**	0.906	0.908	**0.808**	**0.935**
mlplasmids	X	–	0.896	0.273	**0.957**	0.480	0.641
PlasClass	–	–	0.892	0.381	0.939	0.617	0.794
PlasForest	–	X	n/a	0.486	0.939	0.711	0.852
Platon	–	X	n/a	**1**	0.5	0.667	0.924
Deeplasmid	–	X	n/a	n/a	n/a	n/a	n/a
RFPlasmid	X	X	0.973	0.854	0.789	0.667	0.885
**B: Chromosome classification, contigs** >**100 bp**, *n*=**38,110**							
plASgraph2	–	–	**0.991**	0.975	**1**	0.968	0.943
mlplasmids	X	–	0.908	**1**	0.540	0.697	0.609
PlasClass	–	–	0.878	**1**	0.738	0.840	0.766
PlasForest	–	X	n/a	0.992	0.771	0.855	0.795
Platon	–	X	n/a	0.957	**1**	**0.973**	**0.952**
Deeplasmid	–	X	n/a	n/a	n/a	n/a	n/a
RFPlasmid	X	X	0.959	0.982	0.936	0.933	0.893
**C: Plasmid classification, contigs** >**1,000 bp**, *n*=**15,687**							
plASgraph2	–	–	0.997	0.960	0.933	0.852	0.946
mlplasmids	X	–	0.974	0.526	**1**	0.783	0.864
PlasClass	–	–	0.986	0.75	**1**	0.857	0.929
PlasForest	–	X	n/a	0.824	0.944	0.835	0.927
Platon	–	X	n/a	**1**	0.836	**0.897**	**0.961**
Deeplasmid	–	X	0.929	**1**	0.333	0.5	0.892
RFPlasmid	X	X	**0.998**	0.914	0.926	0.862	0.942
**D: Chromosome classification, contigs** >**1,000 bp**, *n*=**15,687**							
plASgraph2	–	–	**0.996**	0.976	**1**	0.969	0.951
mlplasmids	X	–	0.966	**1**	0.845	0.906	0.860
PlasClass	–	–	0.972	**1**	0.897	0.936	0.904
PlasForest	–	X	n/a	**1**	0.919	0.936	0.902
Platon	–	X	n/a	0.989	**1**	**0.983**	**0.973**
Deeplasmid	–	X	0.911	0.903	**1**	0.935	0.893
RFPlasmid	X	X	0.987	**1**	0.954	0.954	0.931

The table shows the median values for each metric from results on all 224 samples included in the testing set. The highest value in each category is shown in bold. SS, method uses Species-Specific models; DB, method uses a DataBase of plasmids and/or chromosomes or other features derived from homology search. Note that, Deeplasmid only allows classification of contigs longer than 1,000 bp. Further, PlasForest and Platon do not provide confidence scores for each prediction. Therefore, calculation of AUROC is not applicable (n/a).

When we restrict the evaluation to contigs longer than 1 kbp (Tables 1C, D), the advantages of homology-based tools (notably Platon and RFPlasmid) become more apparent, as these approaches, as expected, work better on longer contigs. However, plASgraph2 achieves only slightly lower F1 score and accuracy and still outperfoms Platon and RFPlasmid in some of the performance measures.

**Figure 2:**
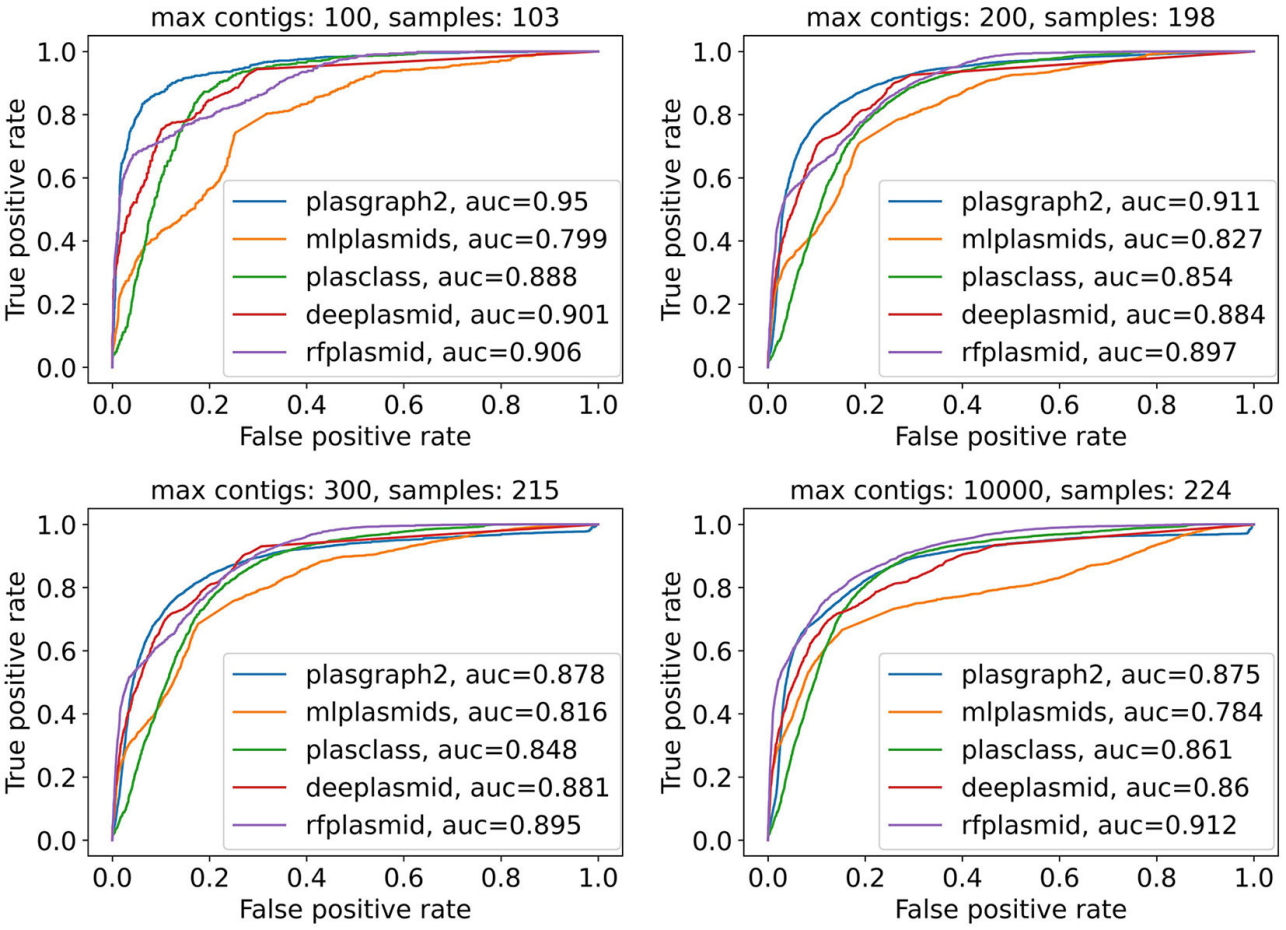
Receiver operating characteristic curves for all contigs in the ESKAPEE test set considering isolates with maximally 100, 200, 300, or 10,000 contigs. ROC curves are not calculated for Platon and PlasForest tools, as those tools do not provide confidence scores as output. In total, the ESKAPEE test set consists of 224 samples; thus almost half of those short read assemblies contain 100 or fewer contigs.

Finally, Figure 2 and Supplementary Figure 2 show that plASgraph2 accuracy is higher on assembly graphs with a lower number of contigs. Large number of contigs in the assembly graph often indicates problems with the underlying data, for example, sample contamination. The performance of all classification methods diminishes on larger assembly graphs, but plASgraph2 seems to be more sensitive to the assembly quality than RFPlasmid or Deeplasmid. Regardless, plASgraph2 outperforms all the other methods on assemblies with up to 200 contigs, which represent a wide majority in our testing set (198 out of 224).

In our evaluation, we consider a prediction of an ambiguous contig as positive for both chromosome and plasmid labels. This may give an undue advantage to plASgraph2, since it can use ambiguous labels even for those contigs where the results are inconclusive, thus securing positive points in at least one of the prediction tasks. To ascertain whether this advantage impacted the results, we have employed two other evaluation measures, forcing the problem to become a standard two-way classification (Supplementary Table 3) and treating the problem as a single three-way classification (Supplementary Table 4). In both cases, the general trends described above remain unchanged.

### 3.3. Evaluation on the non-ESKAPEE data sets

Beyond the ESKAPEE data set evaluation, we also considered testing samples from several non-ESKAPEE bacterial species evolutionarily close to those in the training set: *Citrobacter freundii, Escherichia fergusonii, Klebsiella oxytoca*, and *Salmonella enterica* (see Supplementary Table 1). These species belong to the Enterobacteriaceae family together with three species included in the training set. In total, the samples contained 39,007 contigs of length greater than 100 with known label, of which 10,018 contigs were plasmidic or ambiguous. We excluded mlplasmids from the comparison as it is species-specific and does not have trained models for these species. Furthermore, the Deeplasmid method is not shown as it is unable to classify contigs shorter than 1 kbp. All other methods included data from these species in the databases they used either for training an ML model or for the classification. In contrast, the training set for plASgraph2 did not contain any data from non-ESKAPEE species.

**Figure 3:**
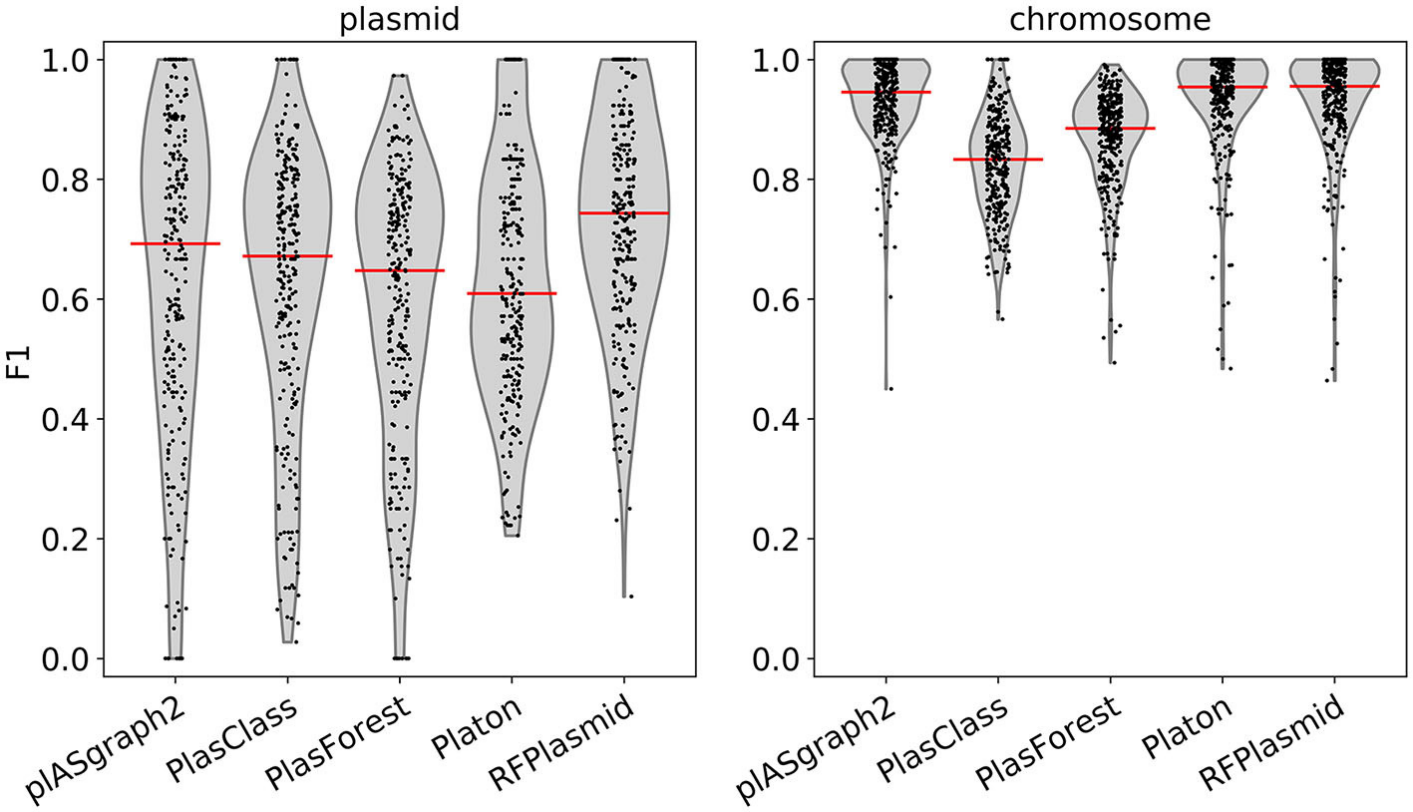
Comparison of F1-scores using samples of evolutionarily close non-ESKAPEE species considering all contigs longer than 100 bp. Each datapoint represents the F1-score of a single isolate. Median is shown as a horizontal line.

Figure 3 shows the F1-score distribution over all considered samples, for both the chromosome and plasmid classification tasks. We observe that plASgraph2, Platon, and RFPlasmid showed the highest chromosomal contig classification F1-scores across this data set, whereas for the plasmid classification, plASgraph2 was the second best performing method after RFPlasmids. A breakdown per species of the results, as shown in Figure 3, is shown in Supplementary Figure 5. Among these methods, plASgraph2 is the only approach that is independent of plasmid database homology features and does not include data from the considered species in its training data set. This experiment shows that plASgraph2 design can successfully generalize to closely related species, not directly included in the training set.

The generalization properties, however, do not extend to more distant non-ESKAPEE species. In particular, we have tested plASgraph2 on *Mycolicibacteriae* spp., *Campylobacter jejuni*, and *Bacillus* spp. (see Supplementary Table 1). Supplementary Figure 6 shows that while in chromosome classification task, the plASgraph2 performance is still comparable to other tools, the plasmid classification does not work very well. None of the tools work well on *Mycolicibacterium*, while Platon and RFPlasmid perform well on *C. jejuni* and PlasForest and Platon on *Bacillus* spp. Additional analysis (see Supplementary Figure 7) showed that while *K. oxytoca* (a representative of species close to ESKAPEE) shows distinct differences between chromosome and plasmid contigs in *k*-mer composition, GC-content, and multiplicity, each of the three distant data sets has at least some of these characteristics almost indistinguishable between plasmids and chromosomes. *C. jejuni* is the most extreme example, where none of these features can be effectively used to distinguish plasmids from chromosomes. Thus, these species present a very difficult case for tools such as plASgraph2 that base their predictions exclusively on sequence features.

**Figure 4:**
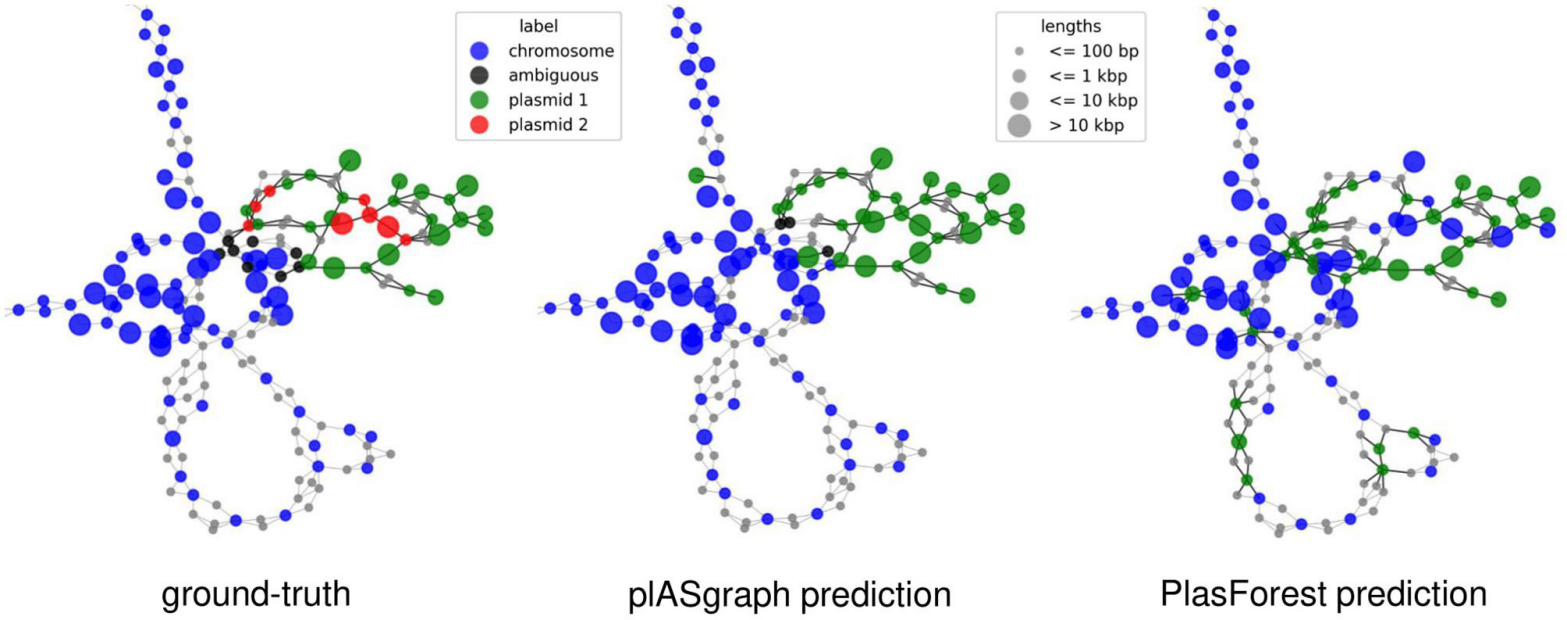
Contig classification in the context of the assembly graph of *C. freundii* isolate SAMN15148288. Chromosomal contigs are colored in blue and ambiguous contigs are colored in black. **(Left)** The ground-truth, including two different plasmids (green and red). **(Middle)** plASgraph2 predictions. **(Right)** PlasForest predictions. Note that, the classification tasks do not include binning of contig plasmids, thus all predicted plasmid contigs are colored in green. The assembly graph extends to the upper left as a loop of chromosomal contigs alternating with unlabeled SNPs, which is not shown.

Finally, plASgraph2 not only provides a score for plasmidic and chromosomal contigs, but also outputs a visualization of an assembly graph labeled according to the predictions. Figure 4 shows parts of the assembly graph for *C. freundii* isolate SAMN15148288 with nodes colored according to the ground truth and both plASgraph2 and PlasForest predictions. The ground truth supports our initial reasoning to incorporate the information provided in the assembly graph, as linked contigs are more likely to belong to the same class. While both tools make some incorrect predictions, visualization clearly shows several isolated chromosome predictions among plasmid contigs and vice versa in the PlasForest prediction, whereas plASgraph2 has only one such isolated false positive.

## 4. Discussion and conclusion

PlASgraph2 is an ML tool designed to identify plasmidic, chromosomal, and ambiguous contigs directly from a bacterial assembly graph using a GNN architecture. Our tool is easy to use, as it only requires a short-read assembly graph file as input, and outperforms other state-of-the-art methods on ESKAPEE species and other related pathogens, especially when including short contigs (<1 kbp) in the evaluation, while obtaining comparable results with contigs above 1 kbp. The performance of plASgraph2 is especially noteworthy considering the potential for the classification of unknown plasmids, as, once a model has been trained, performing classification using plASgraph2 is completely independent of sequence homology. This feature is an important step toward the identification of previously unknown plasmids, which can be critical for diverse One-Health epidemiologic surveillance (Cox et al., 2021). Additionally, plASgraph2 is not dependent on specific species and can therefore be used for newly sequenced bacteria for which no closed genome sequence is available yet.

On contigs longer than 1kb, Platon was the best performing method on the ESKAPEE test set. This result suggests that the assembly graph information can be complemented by homology information to enable better performance. However, accurate classification of shorter contigs by plASgraph2 may enable identification of more complete plasmids from incomplete assemblies and has the potential to facilitate novel plasmid discovery.

Another novel feature of plASgraph2 is the separation of plasmid and chromosome classification tasks, recognizing that some contigs are ambiguous, being parts of both types of molecules. These ambiguous contigs are an interesting subject for further study by themselves; our preliminary analysis of ambiguous contigs in our data sets suggests that the majority of them are related to transposons and phages. These mobile elements can integrate into both plasmids and chromosomes within the cell.

The simplicity of the architecture of the plASgraph2 model makes it amenable to extensions. For example, the use of additional information about plasmids, such as the presence of plasmid-specific genes in a contig, could allow for further increase in classification accuracy as this additional information would propagate to nearby nodes due to the GNN architecture.

## Data availability statement

Publicly available data sets were analyzed in this study. They were downloaded from the GenBank database. The list of accession numbers as well as processed training and testing data sets are available at https://github.com/fmfi-compbio/plasgraph2-datasets. Our software is available at https://github.com/cchauve/plasgraph2.

## Author contributions

JS: Data curation, Investigation, Methodology, Software, Visualization, Writing—review and editing. KS: Investigation, Methodology, Writing—review and editing. BB: Conceptualization, Data curation, Investigation, Methodology, Software, Supervision, Validation, Writing—original draft, Writing—review and editing. TV: Conceptualization, Data curation, Investigation, Methodology, Software, Supervision, Validation, Writing—original draft, Writing—review and editing. CC: Conceptualization, Data curation, Investigation, Methodology, Software, Supervision, Writing—original draft, Writing—review and editing.

## References

[B1] Abadi M. Agarwal A. Barham P. Brevdo E. Chen Z. Citro C. (2015). TensorFlow: Large-Scale Machine Learning on Heterogeneous Systems. Available online at: tensorflow.org

[B2] Acman M. Wang R. van Dorp L. Shaw L. P. Wang Q. Luhmann N.. (2022). Role of mobile genetic elements in the global dissemination of the carbapenem resistance gene bla_*NDM*_. Nat. Commun. 13, 1131. 10.1038/s41467-022-28819-2 35241674 PMC8894482

[B3] Andreopoulos W. B. Geller A. M. Lucke M. Balewski J. Clum A. Ivanova N. N.. (2021). Deeplasmid: deep learning accurately separates plasmids from bacterial chromosomes. Nucleic Acids Res. 50, e17. 10.1093/nar/gkab1115 34871418 PMC8860608

[B4] Arredondo-Alonso S. Bootsma M. Hein Y. Rogers M. R. C. Corander J. Willems R. J. L.. (2020). gplas: a comprehensive tool for plasmid analysis using short-read graphs. Bioinformatics 36, 3874–3876. 10.1093/bioinformatics/btaa233 32271863 PMC7320608

[B5] Arredondo-Alonso S. Rogers M. R. Braat J. C. Verschuuren T. D. Top J. Corander J.. (2018). mlplasmids: a user-friendly tool to predict plasmid-and chromosome-derived sequences for single species. Microb. Genom. 4, e000224. 10.1099/mgen.0.000224 30383524 PMC6321875

[B6] Arredondo-Alonso S. Willems R. J. van Schaik W. Schürch A. C. (2017). On the (im)possibility of reconstructing plasmids from whole-genome short-read sequencing data. Microb. Genom. 3, e000128. 10.1099/mgen.0.000128 29177087 PMC5695206

[B7] Bankevich A. Nurk S. Antipov D. Gurevich A. A. Dvorkin M. Kulikov A. S.. (2012). SPAdes: a new genome assembly algorithm and its applications to single-cell sequencing. J. Comput. Biol. 19, 455–477. 10.1089/cmb.2012.0021 22506599 PMC3342519

[B8] Boostrom I. Portal E. A. Spiller O. B. Walsh T. R. Sands K. (2022). Comparing long-read assemblers to explore the potential of a sustainable low-cost, low-infrastructure approach to sequence antimicrobial resistant bacteria with Oxford Nanopore sequencing. Front. Microbiol. 13, 796465. 10.3389/fmicb.2022.796465 35308384 PMC8928191

[B9] Chan A. P. Choi Y. Clarke T. H. Brinkac L. M. White R. C. Jacobs M. R.. (2020). AbGRI4, a novel antibiotic resistance island in multiply antibiotic-resistant *Acinetobacter baumannii* clinical isolates. J. Antimicrob. Chemother. 75, 2760–2768. 10.1093/jac/dkaa266 32681170 PMC7556812

[B10] Chen Z. Kuang D. Xu X. Gonzalez-Escalona N. Erickson D. L. Brown E.. (2020). Genomic analyses of multidrug-resistant *Salmonella Indiana, Typhimurium*, and *Enteritidis* isolates using MinION and MiSeq sequencing technologies. PLoS ONE 15, e0235641. 10.1371/journal.pone.0235641 32614888 PMC7332006

[B11] Chollet F. (2015). Keras. Available online at: https://keras.io

[B12] Cox G. W. Parmley E. J. Avery B. P. Irwin R. J. Reid-Smith R. J. Deckert A. E.. (2021). A one-health genomic investigation of gentamicin resistance in *Salmonella* from human and chicken sources in Canada, 2014 to 2017. Antimicrob. Agents Chemother. 65, e0096621. 10.1128/AAC.00966-21 34570642 PMC8597779

[B13] De Oliveira D. M. P. Forde B. M. Kidd T. J. Harris P. N. A. Schembri M. A. Beatson S. A.. (2020). Antimicrobial resistance in ESKAPE pathogens. Clin. Microbiol. Rev. 33, e00181-19. 10.1128/CMR.00181-19 32404435 PMC7227449

[B14] Fang Z. Tan J. Wu S. Li M. Xu C. Xie Z.. (2019). PPR-Meta: a tool for identifying phages and plasmids from metagenomic fragments using deep learning. GigaScience 8, giz066. 10.1093/gigascience/giz066 31220250 PMC6586199

[B15] Furuta Y. Tsujinouchi M. Shawa M. Zorigt T. Miyajima Y. Paudel A.. (2022). Complete genome sequences of 24 strains of *Bacillus cereus* isolated from nosocomial infection and bacteremia cases in Japan. Microbiol. Resour. Announc. 11, e01203-21. 10.1128/mra.01203-21 35289651 PMC9022585

[B16] Grattarola D. Alippi C. (2021). Graph neural networks in TensorFlow and Keras with Spektral [application notes]. IEEE Comput. Intell. Mag. 16, 99–106. 10.1109/MCI.2020.3039072

[B17] Hikichi M. Nagao M. Murase K. Aikawa C. Nozawa T. Yoshida A.. (2019). Complete genome sequences of eight methicillin-resistant *Staphylococcus aureus* strains isolated from patients in Japan. Microbiol. Resour. Announc. 8, 10–1128. 10.1128/MRA.01212-19 31753944 PMC6872886

[B18] Johnson S. L. Daligault H. E. Davenport K. W. Jaissle J. Frey K. G. Ladner J. T.. (2015). Complete genome sequences for 35 biothreat assay-relevant Bacillus species. Genome Announc. 3, 10–1128. 10.1128/genomeA.00151-15 25931591 PMC4417687

[B19] Kiesewalter H. T. Lozano-Andrade C. N. Maróti G. Snyder D. Cooper V. S. Jørgensen T. S.. (2020). Complete genome sequences of 13 *Bacillus subtilis* soil isolates for studying secondary metabolite diversity. Microbiol. Resour. Announc. 9, 10–1128. 10.1128/MRA.01406-19 31919181 PMC6952667

[B20] Kingma D. P. Ba J. (2014). Adam: a method for stochastic optimization. arXiv preprint arXiv:1412.6980. 10.48550/arXiv.1412.6980

[B21] Kipf T. N. Welling M. (2016). Semi-supervised classification with graph convolutional networks. arXiv preprint arXiv:1609.02907. 10.48550/arXiv.1609.02907

[B22] Krawczyk P. S. Lipinski L. Dziembowski A. (2018). PlasFlow: predicting plasmid sequences in metagenomic data using genome signatures. Nucleic Acids Res. 46, e35. 10.1093/nar/gkx1321 29346586 PMC5887522

[B23] Lamurias A. Sereika M. Albertsen M. Hose K. Nielsen T. D. (2022). Metagenomic binning with assembly graph embeddings. bioRxiv. 38, 4481–4487. 10.1093/bioinformatics/btac557 35972375 PMC9525014

[B24] Li H. (2018). Minimap2: pairwise alignment for nucleotide sequences. Bioinformatics 34, 3094–3100. 10.1093/bioinformatics/bty191 29750242 PMC6137996

[B25] Magalhães B. Senn L. Blanc D. S. (2019). High-quality complete genome sequences of three *Pseudomonas aeruginosa* isolates retrieved from patients hospitalized in intensive care units. Microbiol. Resour. Announc. 8, 10–1128. 10.1128/MRA.01624-18 30834388 PMC6395873

[B26] Matsumoto Y. Kinjo T. Motooka D. Nabeya D. Jung N. Uechi K.. (2019). Comprehensive subspecies identification of 175 nontuberculous mycobacteria species based on 7547 genomic profiles. Emerg. Microb. Infect. 8, 1043–1053. 10.1080/22221751.2019.1637702 31287781 PMC6691804

[B27] Morita D. Arai H. Isobe J. Maenishi E. Kumagai T. Maruyama F.. (2023). Whole-genome and plasmid comparative analysis of *Campylobacter jejuni* from human patients in Toyama, Japan, from 2015 to 2019. Microbiol. Spectrum 11, e02659-22. 10.1128/spectrum.02659-22 36622198 PMC9927224

[B28] Ono H. K. Suzuki Y. Kubota H. Asano K. Takai S. Nakane A.. (2021). Complete genome sequence of *Staphylococcus aureus* strain 834, isolated from a septic patient in Japan. Microbiol. Resour. Announc. 10, 10–1128. 10.1128/MRA.01477-20 33664145 PMC7936643

[B29] Partridge S. R. Kwong S. M. Firth N. Jensen S. O. (2018). Mobile genetic elements associated with antimicrobial resistance. Clin. Microbiol. Rev. 31, e00088-17. 10.1128/CMR.00088-17 30068738 PMC6148190

[B30] Pellow D. Mizrahi I. Shamir R. (2020). PlasClass improves plasmid sequence classification. PLoS Comput. Biol. 16, e1007781. 10.1371/journal.pcbi.1007781 32243433 PMC7159247

[B31] Pellow D. Zorea A. Probst M. Furman O. Segal A. Mizrahi I.. (2021). SCAPP: an algorithm for improved plasmid assembly in metagenomes. Microbiome 9, 144. 10.1186/s40168-021-01068-z 34172093 PMC8228940

[B32] Peter S. Bosio M. Gross C. Bezdan D. Gutierrez J. Oberhettinger P.. (2020). Tracking of antibiotic resistance transfer and rapid plasmid evolution in a hospital setting by nanopore sequencing. mSphere 5, e00525-20. 10.1128/mSphere.00525-20 32817379 PMC7440845

[B33] Pradier L. Tissot T. Fiston-Lavier A.-S. Bedhomme S. (2021). PlasForest: a homology-based random forest classifier for plasmid detection in genomic datasets. BMC Bioinformatics 22, 349. 10.1186/s12859-021-04270-w 34174810 PMC8236179

[B34] Pu L. Shamir R. (2022). 3CAC: improving the classification of phages and plasmids in metagenomic assemblies using assembly graphs. bioRxiv. 38, ii56–ii61. 10.1093/bioinformatics/btac468 36124804

[B35] Robertson J. Nash J. (2018). MOB-suite: software tools for clustering, reconstruction and typing of plasmids from draft assemblies. Microb. Genom. 4, e000206. 10.1099/mgen.0.000206 30052170 PMC6159552

[B36] Schmartz G. P. Hartung A. Hirsch P. Kern F. Fehlmann T. Müller R.. (2022). PLSDB: advancing a comprehensive database of bacterial plasmids. Nucleic Acids Res. 50, D273–D278. 10.1093/nar/gkab1111 34850116 PMC8728149

[B37] Schwengers O. Barth P. Falgenhauer L. Hain T. Chakraborty T. Goesmann A. (2020). Platon: identification and characterization of bacterial plasmid contigs in short-read draft assemblies exploiting protein sequence-based replicon distribution scores. Microb. Genom. 6, e000398. 10.1099/mgen.0.000398 32579097 PMC7660248

[B38] Shaw L. P. Chau K. K. Kavanagh J. AbuOun M. Stubberfield E. Gweon H. S.. (2021). Niche and local geography shape the pangenome of wastewater-and livestock-associated *Enterobacteriaceae*. Sci. Adv. 7, eabe3868. 10.1126/sciadv.abe3868 33837077 PMC8034854

[B39] Souvorov A. Agarwala R. Lipman D. J. (2018). SKESA: strategic k-mer extension for scrupulous assemblies. Genome Biol. 19, 153. 10.1186/s13059-018-1540-z 30286803 PMC6172800

[B40] van der Graaf-van Bloois L. Wagenaar J. A. Zomer A. L. (2021). RFPlasmid: predicting plasmid sequences from short-read assembly data using machine learning. Microb. Genom. 7, 000683. 10.1099/mgen.0.000683 34846288 PMC8743549

[B41] Wick R. R. Judd L. M. Gorrie C. L. Holt K. E. (2017). Unicycler: resolving bacterial genome assemblies from short and long sequencing reads. PLoS Comput. Biol. 13, e1005595. 10.1371/journal.pcbi.1005595 28594827 PMC5481147

[B42] Zhou F. Xu Y. (2010). cBar: a computer program to distinguish plasmid-derived from chromosome-derived sequence fragments in metagenomics data. Bioinformatics 26, 2051–2052. 10.1093/bioinformatics/btq299 20538725 PMC2916713

