# Supplementary material for: plASgraph2: using graph neural networks to detect plasmid contigs from an assembly graph
Source: Front Microbiol. 2023 Oct 6;14:1267695. doi: 10.3389/fmicb.2023.1267695 (PMC10587606; doi:10.3389/fmicb.2023.1267695)
Supplement: Supplementary file 1 [file Data_Sheet_1.PDF]

frontiers

Supplementary Material

Table S1. Training and testing data sets. Number and total length of contigs with different labels. Only contigs with length greater than 100bp are shown.

Species	Samples	Chromosome		Plasmid		Ambig.		Unknown	
	#	#	Mbp	#	Mbp	#	Mbp	#	Mbp
**ESKAPEE training data**									
ALL	140	25231	595.2	3845	22.7	1450	1.0	3722	21.1
(%)	140	73.7%	93.0%	11.2%	3.5%	4.2%	0.2%	10.9%	3.3%
Enterococcus faecium	20	3019	51.8	1191	4.5	620	0.3	167	0.2
Staphylococcus aureus	20	1578	55.8	52	0.4	13	0.0	21	0.0
Klebsiella pneumoniae	20	5991	101.0	1209	6.8	441	0.2	1773	4.0
Acinetobacter baumannii	20	5238	74.8	747	3.8	70	0.0	748	1.3
Pseudomonas aeruginosa	20	5126	134.2	80	1.1	35	0.2	584	2.4
Enterobacter spp.	20	1991	81.8	328	3.2	178	0.1	410	13.1
Escherichia coli	20	2288	95.8	238	3.0	93	0.1	19	0.0
**ESKAPEE testing data**									
ALL	224	32761	936.8	3970	32.6	1379	0.8	5004	66.6
(%)	224	76.0%	90.4%	9.2%	3.1%	3.2%	0.1%	11.6%	6.4%
Enterococcus faecium	4	361	10.0	105	0.9	67	0.0	22	0.0
Staphylococcus aureus	62	3924	168.6	194	1.4	46	0.0	391	3.9
Klebsiella pneumoniae	92	21720	453.8	2587	21.8	984	0.6	3020	33.9
Acinetobacter baumannii	10	1254	46.9	156	1.2	47	0.0	42	0.5
Pseudomonas aeruginosa	10	1165	65.4	0	0.0	0	0.0	110	1.6
Enterobacter spp.	30	1861	123.5	358	4.3	100	0.1	660	17.1
Escherichia coli	16	2476	68.6	570	3.1	135	0.1	759	9.5
**Non-ESKAPEE testing data, close**									
ALL	320	28989	1518.2	7901	65.3	2117	2.1	2993	71.0
(%)	320	69.0%	91.6%	18.8%	3.9%	5.0%	0.1%	7.1%	4.3%
Citrobacter freundii	100	8314	478.8	2227	16.8	986	0.7	512	13.0
Escherichia fergusonii	100	7234	415.6	1077	14.9	173	0.2	1350	41.5
Klebsiella oxytoca	62	6923	348.7	3463	23.8	753	0.7	825	15.4
Salmonella enterica	58	6518	275.0	1134	9.8	205	0.6	306	1.1
**Non-ESKAPEE testing data, distant**									
ALL	188	46260	775.9	4260	30.7	445	0.7	2031	10.3
(%)	188	87.3%	94.9%	8.0%	3.8%	0.8%	0.1%	3.8%	1.3%
Mycolicibacteriae	60	6296	338.3	222	6.2	59	0.1	372	4.6
Campylobacter jejuni	60	3521	100.8	238	3.7	23	0.0	86	0.3
Bacillus spp.	68	36443	336.8	3800	20.9	363	0.6	1573	5.4

Table S2. Reference genomes for the homology-based gold-standard labeling

Accession	Description
NZ_CP038996.1	Enterococcus faecium strain SRR24
NC_007795.1	Staphylococcus aureus subsp. aureus NCTC 8325
NC_016845.1	Klebsiella pneumoniae subsp. pneumoniae HS11286
NZ_CP043953.1	Acinetobacter baumannii strain K09-14
NC_002516.2	Pseudomonas aeruginosa PAO1
NZ_CP009756.1	Enterobacter cloacae strain GGT036
NC_000913.3	Escherichia coli str. K-12 substr. MG1655
NC_002695.2	Escherichia coli O157:H7 str. Sakai DNA
NZ_AP019632.1	Enterobacter asburiae strain 1808-013
NZ_AP022508.1	Enterobacter bugandensis strain STN0717-56
NZ_CP081105.1	Enterobacter cancerogenus strain JY65
NZ_CP077392.1	Enterobacter hormaechei strain FDAARGOS 1435
NZ_CP017184.1	Enterobacter roggenkampii strain DSM 16690
NZ_CP044060.1	Aeromonas veronii strain FDAARGOS_632
NC_007530.2	Bacillus anthracis str. 'Ames Ancestor'
NC_002163.1	Campylobacter jejuni subsp. jejuni NCTC 11168 = ATCC 700819
NZ_CP033744.1	Citrobacter freundii strain FDAARGOS_549
NC_009495.1	Clostridium botulinum A str. ATCC 3502
NZ_CP057657.1	Escherichia fergusonii strain RHB19-C05
NZ_CP041925.1	Klebsiella aerogenes strain Ka37751
NZ_AP022547.1	Klebsiella michiganensis strain THO-011
NZ_CP033844.1	Klebsiella oxytoca strain FDAARGOS_500
NZ_CP065838.1	Klebsiella quasipneumoniae strain KqPF26
NC_003210.1	Listeria monocytogenes EGD-e
NZ_CP069288.1	Priestia megaterium strain ATCC 14581
NC_003197.2	Salmonella enterica subsp. enterica serovar Typhimurium str. LT2
NC_004603.1	Vibrio parahaemolyticus RIMD 2210633 chromosome 1
NC_004605.1	Vibrio parahaemolyticus RIMD 2210633 chromosome 2
NC_017168.1	Yersinia pestis A1122
NZ_AP022570.1	Mycolicibacterium poriferae strain JCM 12603
NZ_AP022579.1	Mycolicibacterium boenickei strain JCM 15653
NZ_CP023147.1	Mycobacterium marseillense strain FLAC0026
NZ_CP025546.1	Mycobacterium paragordonae strain 49061
NZ_CP034181.1	Mycobacteroides abscessus strain GZ002
NZ_LT906469.1	Mycolicibacter terrae strain NCTC10856 chromosome 1
NC_002163.1	Campylobacter jejuni subsp. jejuni NCTC 11168 = ATCC 700819
NZ_CP046317.1	Campylobacter coli strain FDAARGOS_735
NZ_CP017060.1	Bacillus cereus strain FORC_047
NC_007530.2	Bacillus anthracis str. 'Ames Ancestor'
NC_000964.3	Bacillus subtilis subsp. subtilis str. 168
NZ_CM000753.1	Bacillus thuringiensis serovar berliner ATCC 10792

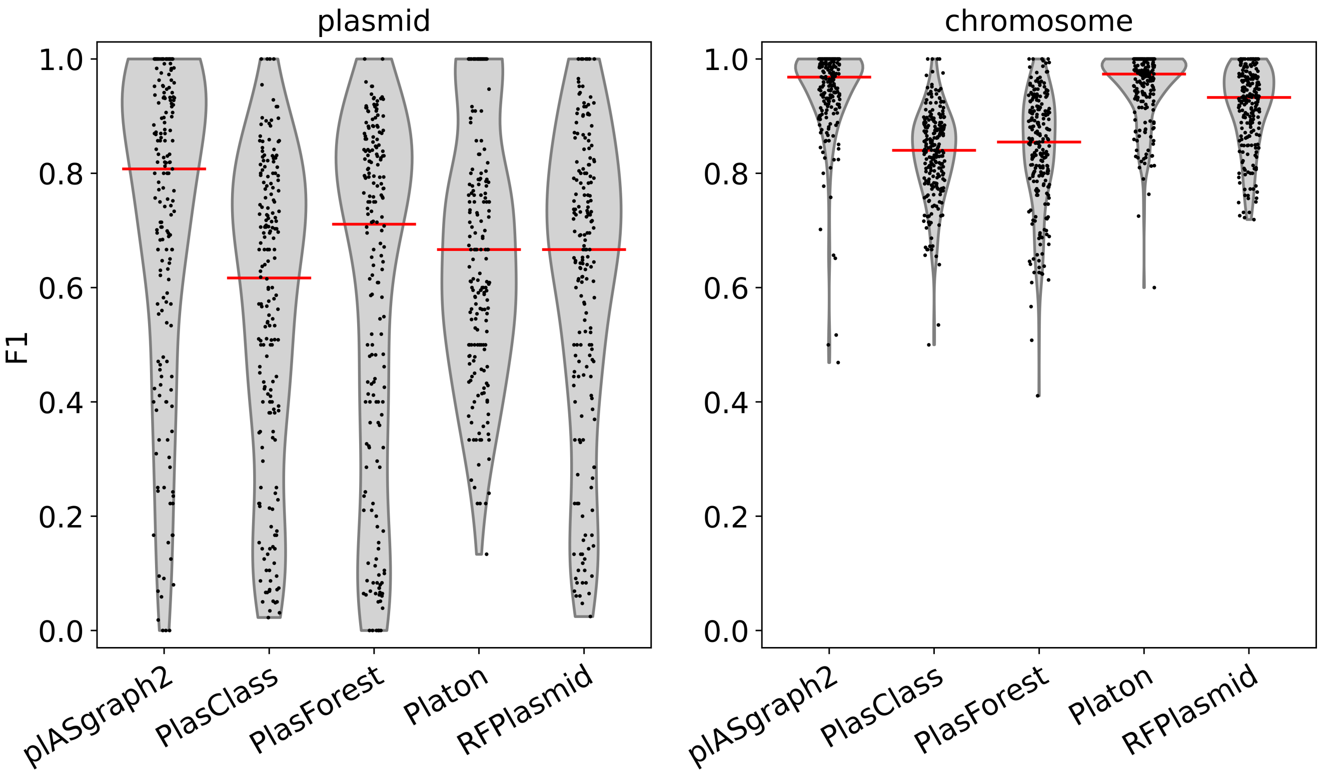

Figure S1. Comparison of F1-scores using samples from ESKAPEE species and considering all contigs longer than 100 bp. Each datapoint represents the F1-score of a single isolate. Median is shown as a horizontal line.

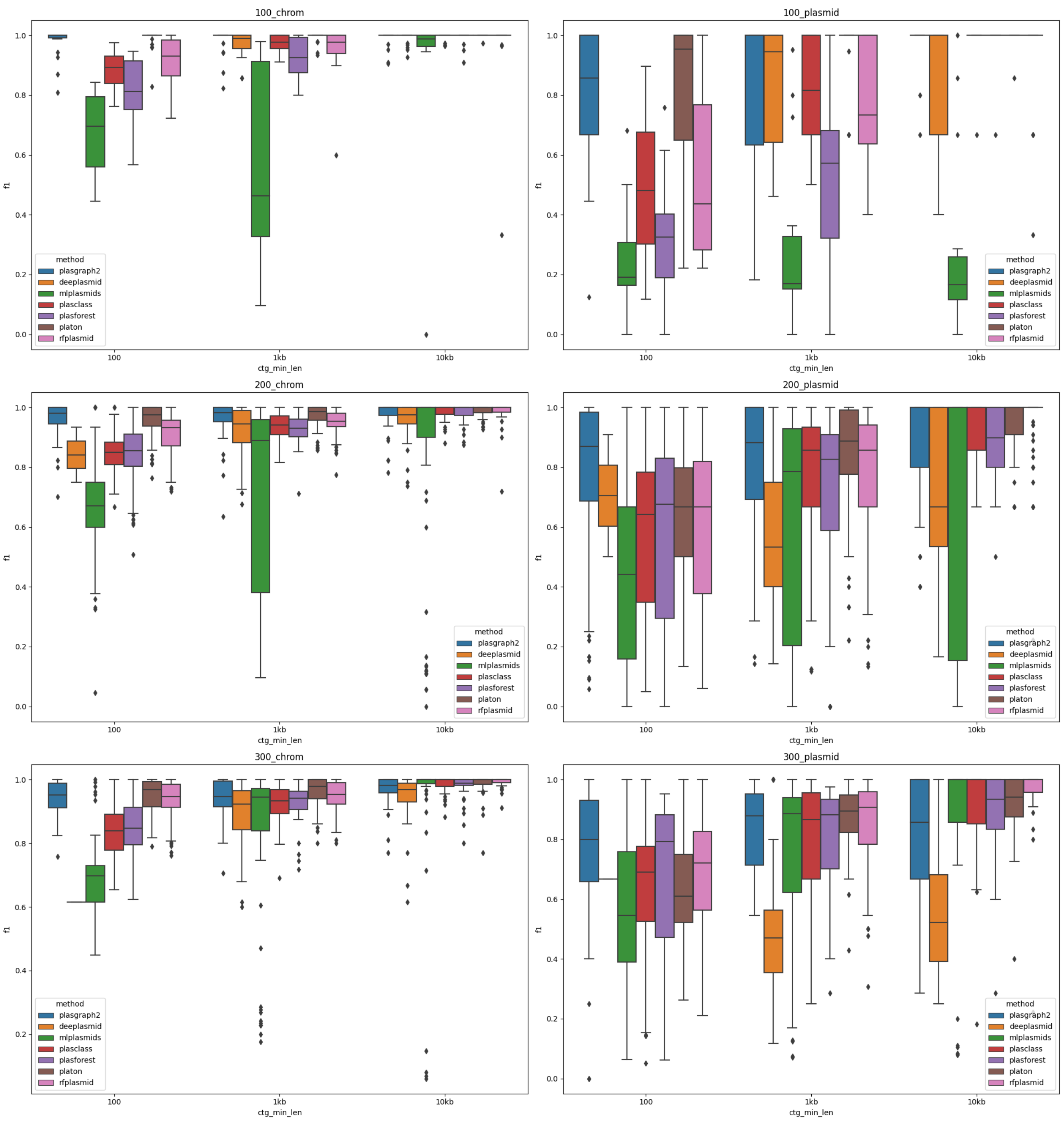

Figure S2. Comparison of F1-scores distribution over all samples for all methods considering only assemblies with a maximum number of 100, 200 or 300 contigs respectively. The plots on the left show the F1-scores for the chromosomal classification and the plots on the right show the results for the plasmid classification. From top to bottom the plots contain all samples that either considered maximally 100, 200 or 300 contigs. In total, only two ESKAPEE samples contained more than 300 contigs and are thus not considered in this figure. Within each plot the comparisons of all methods are shown considering only >100 bp, >1 kbp or >10 kbp contigs from left to right.

Table S3. Performance of contig classification algorithms on the ESKAPEE testing set using strict binary evaluation. The table shows the median values for each metric from among results on samples included in the testing set. The output of plASgraph2 was modified so that "ambiguous" predictions were replaced with the prediction of "plasmid" or "chromosome" depending on which option had a higher score. The contigs with "ambiguous" ground-truth label were removed from this evaluation. Fig. S3 shows the full distribution of F1 scores.

Method	SS	DB	AUROC	Precision	Recall	F1	Accuracy
**A: Plasmid classification,** contigs >100 bp, n =36,731							
plASgraph2	–	–	0.995	0.961	0.900	0.827	0.955
mlplasmids	X	–	0.938	0.235	1.000	0.447	0.627
PlasClass	–	–	0.898	0.333	0.964	0.576	0.796
PlasForest	–	X	n/a	0.451	0.943	0.645	0.851
Platon	–	X	n/a	1.000	0.667	0.772	0.952
RFPlasmid	X	X	0.982	0.833	0.833	0.730	0.910
**B: Chromosome classification**, contigs >100 bp, n =36,731							
plASgraph2	–	–	0.995	0.988	1.000	0.973	0.955
mlplasmids	X	–	0.938	1.000	0.568	0.718	0.627
PlasClass	–	–	0.898	1.000	0.771	0.861	0.796
PlasForest	–	X	n/a	0.992	0.855	0.894	0.851
Platon	–	X	n/a	0.954	1.000	0.973	0.952
RFPlasmid	X	X	0.982	0.982	0.974	0.942	0.907

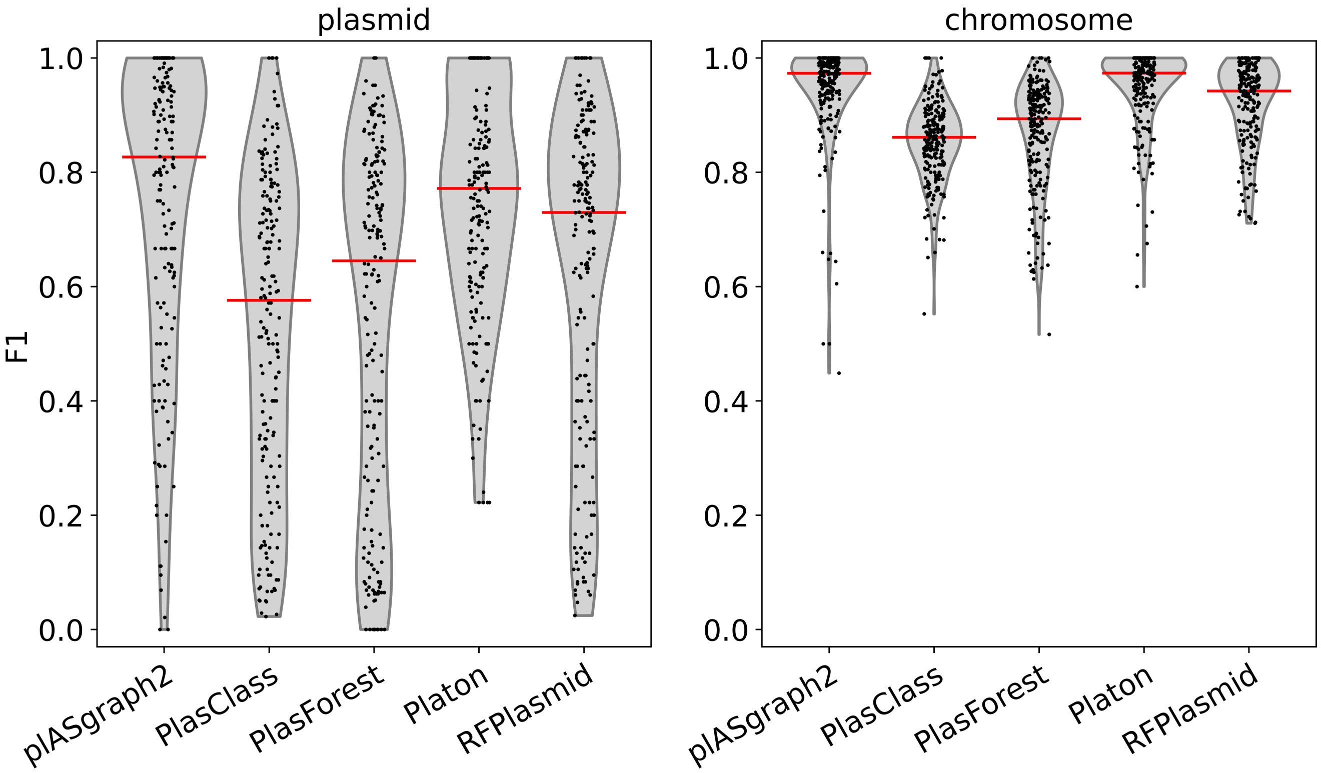

Figure S3. Comparison of F1-scores using samples from ESKAPEE species and considering all contigs longer than 100 bp using strict binary evaluation. Each datapoint represents the F1-score of a single isolate. Median is shown as a horizontal line.

Table S4. Performance of contig classification algorithms on the ESKAPEE testing set using ternary evaluation. The table shows the median values for each metric from among results on samples included in the testing set. Here, ambiguous contigs were considered as a class separate from chromosomes and plasmids. The accuracy of predictions of ambiguous class was evaluated only for plASgraph2, since no other tool predicts ambiguous contigs. Note that while F1-score of plASgraph2 in predicting ambiguous contigs is low, the task is very difficult since it is highly unbalanced (only 3.6% of contigs evaluated in this experiment are ambiguous). Fig. S4 shows the full distribution of F1 scores.

Method	SS	DB	Precision	Recall	F1	Accuracy
**A: Plasmid classification,** contigs >100 bp, n =38,110						
plASgraph2	–	–	1.000	0.775	0.777	0.943
mlplasmids	X	–	0.223	1.000	0.420	0.609
PlasClass	–	–	0.302	0.964	0.522	0.766
PlasForest	–	X	0.333	0.943	0.549	0.795
Platon	–	X	1.000	0.667	0.764	0.952
RFPlasmid	X	X	0.685	0.833	0.667	0.894
**B: Chromosome classification**, contigs >100 bp, n =38,110						
plASgraph2	–	–	0.987	0.984	0.959	0.935
mlplasmids	X	–	0.981	0.568	0.713	0.641
PlasClass	–	–	0.990	0.771	0.852	0.794
PlasForest	–	X	0.986	0.855	0.892	0.852
Platon	–	X	0.923	1.000	0.955	0.924
RFPlasmid	X	X	0.968	0.974	0.929	0.885
**C: Ambiguous classification**, contigs >100 bp, n =38,110						
plASgraph2	–	–	0.167	0.500	0.400	0.941

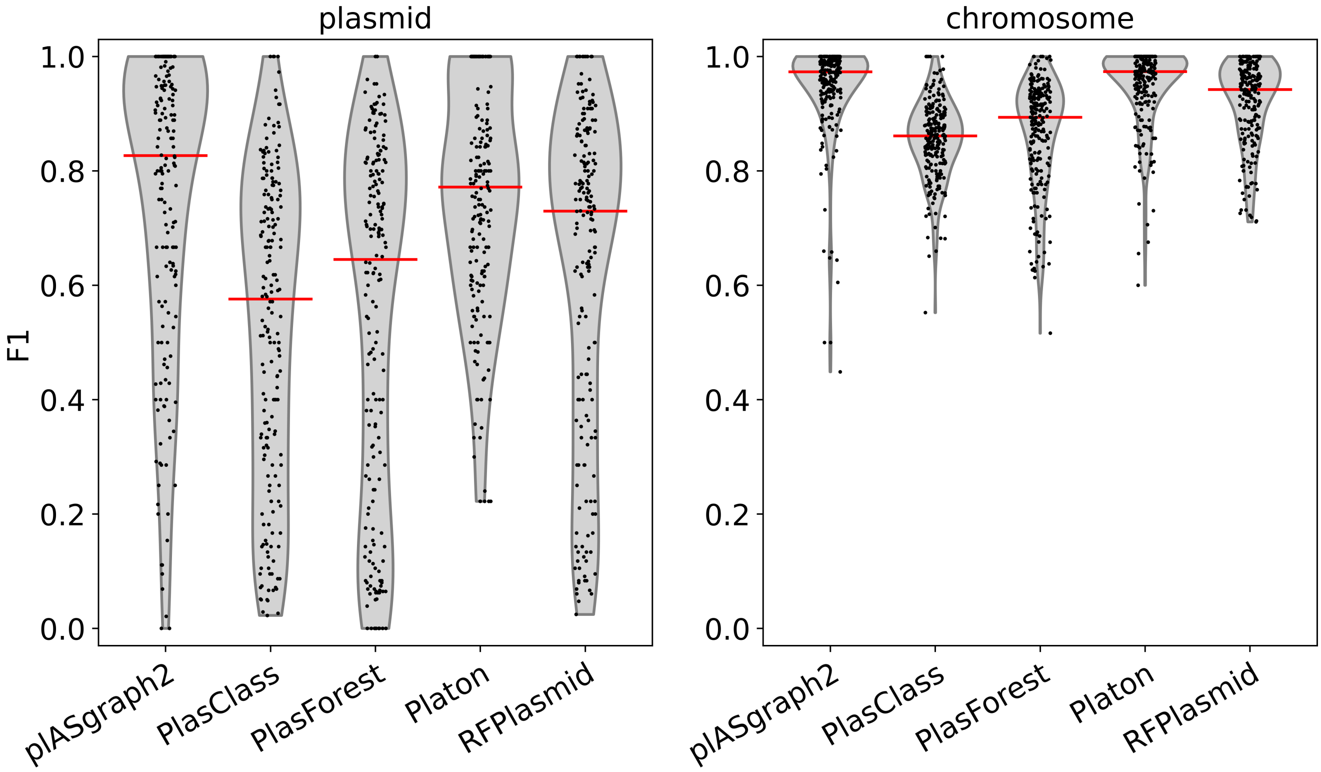

Figure S4. Comparison of F1-scores using samples from ESKAPEE species and considering all contigs longer than 100 bp using ternary evaluation. Each datapoint represents the F1-score of a single isolate. Median is shown as a horizontal line.

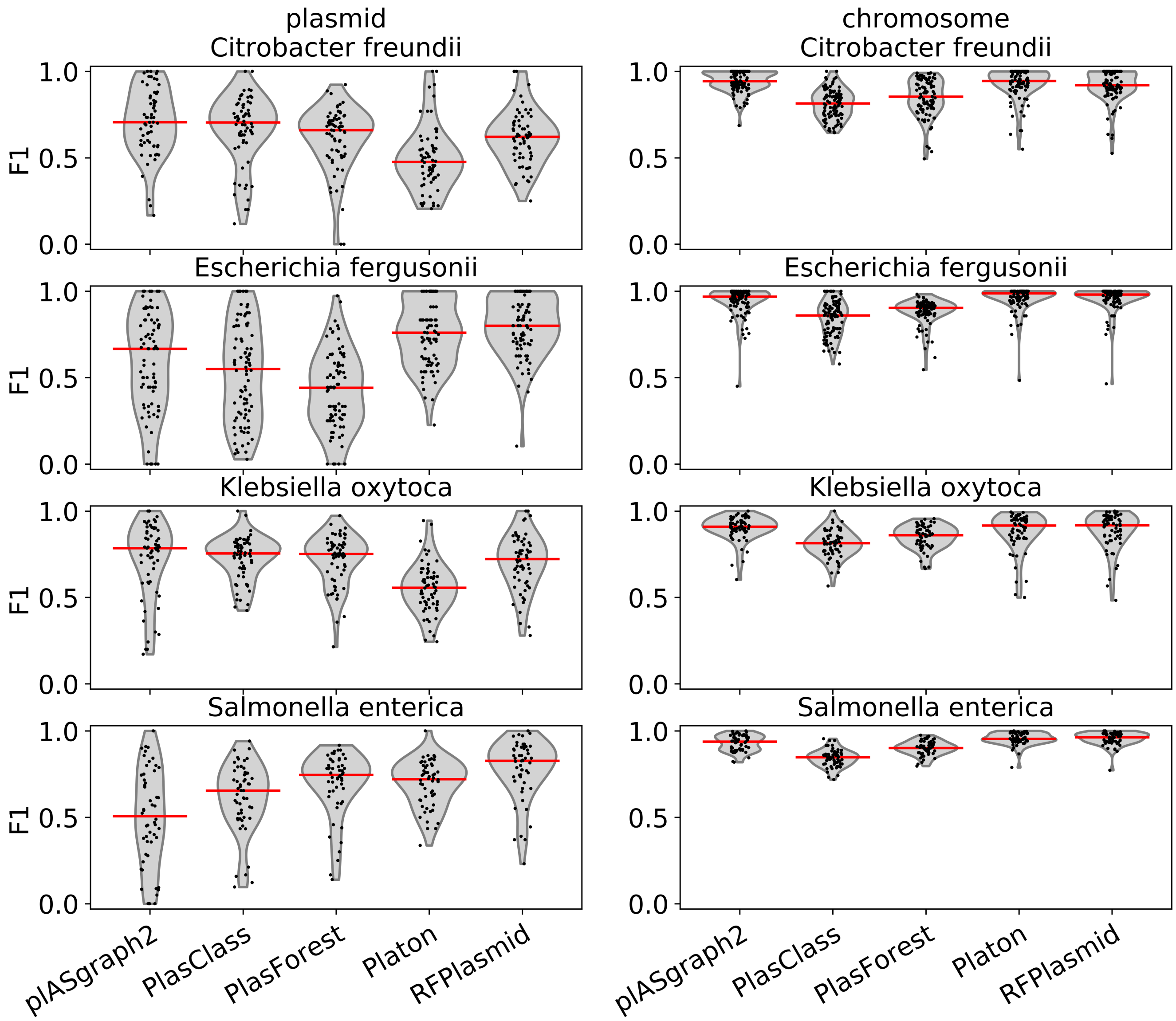

Figure S5. Comparison of F1-scores using samples of close non-ESKAPEE species and considering all contigs longer than 100 bp. Each datapoint represents the F1-score of a single isolate. The horizontal lines represent median.

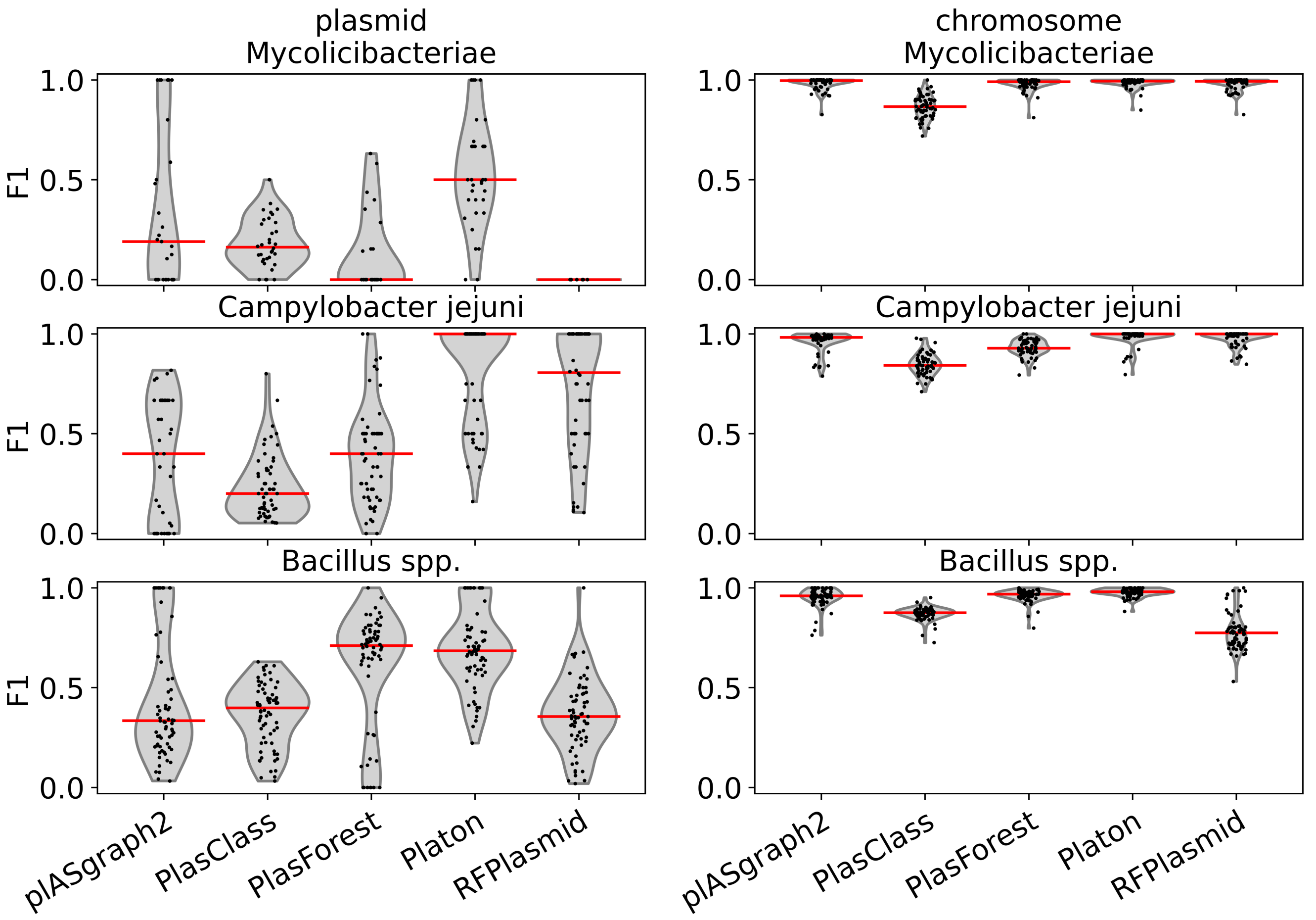

Figure S6. Comparison of F1-scores using samples of more distant non-ESKAPEE species and considering all contigs longer than 100 bp. Each datapoint represents the F1-score of a single isolate. The horizontal lines represent median.

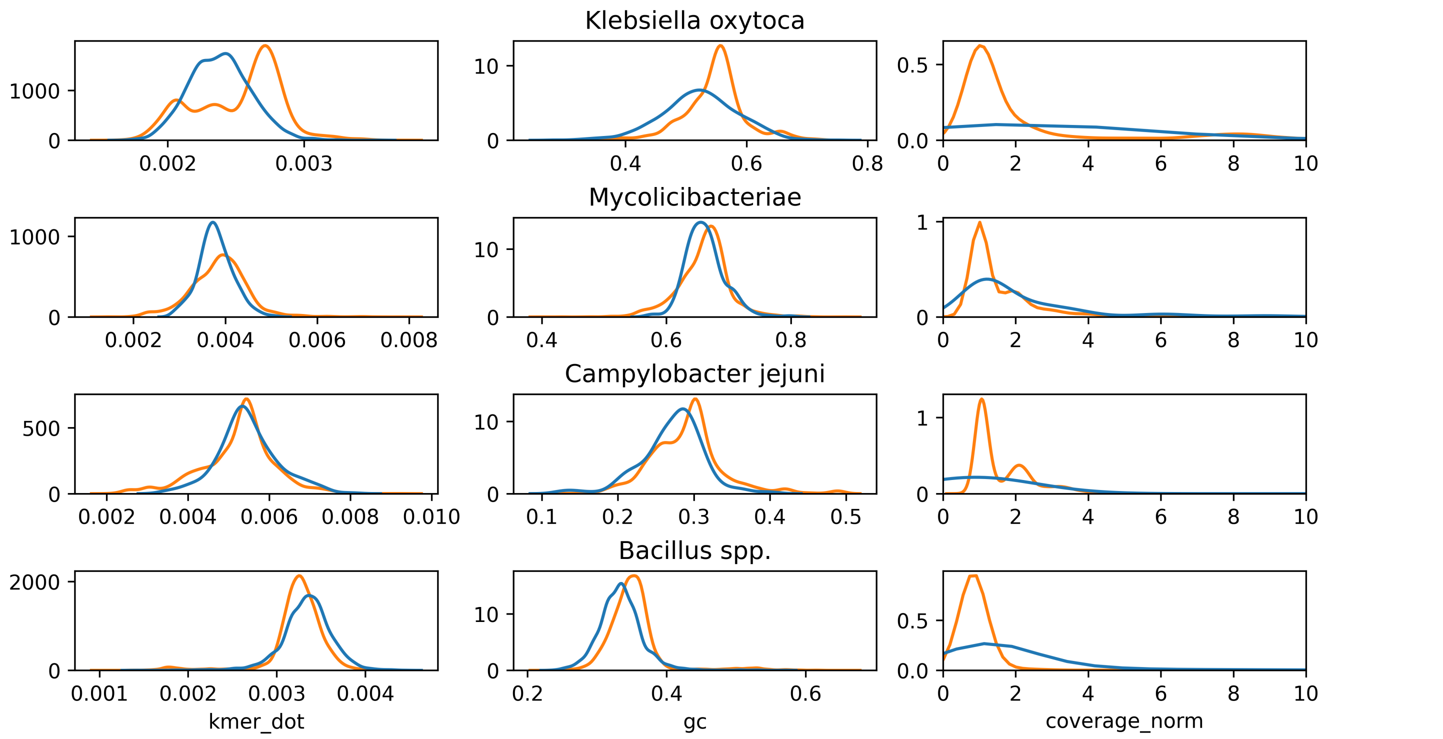

Figure S7. Distribution of sequence-related features of chromosome and plasmid contigs. Orange: chromosome contigs. Blue: plasmid contigs. Ambiguous and unlabeled contigs are not included. Left: relative pentamer content distribution. Middle: GC content distribution. Right: Relative coverage.
